# Baseline vitamin D status is associated with glycemic and weight loss outcomes in patients with type 2 diabetes treated with semaglutide

**DOI:** 10.3389/fnut.2026.1833045

**Published:** 2026-04-27

**Authors:** Ariel Israel, Mahmud Moed, Hala Abo Fanne, Aviv Kedem, Mahmoud Odeh, Kareem Abu Rashed, Tayser Marzook, Isis Abumouch, Rami Abu Fanne

**Affiliations:** 1 Leumit Health Services, Tel Aviv, Israel; 2 Department of Family Medicine, Sackler Faculty of Medicine, Tel-Aviv University, Tel Aviv-Yafo, Israel; 3 Department of Emergency, Hillel Yaffe Medical Center, Hadera, Israel; 4 Department of Medicine, Haemek Medical Center, Afula, Israel; 5 Heart Institute, Hillel Yaffe Medical Center, Hadera, Israel

**Keywords:** lipid control, metabolic indices, semaglutide, vitamin D, weight loss

## Abstract

**Objective:**

Semaglutide, a glucagon-like peptide-1 receptor agonist, is an established therapy for type 2 diabetes (T2D), offering robust glycemic control and weight reduction. Vitamin D has been implicated in metabolic regulation, yet its influence on semaglutide-induced outcomes remains unclear.

**Research design and methods:**

We conducted a retrospective cohort study of 5,384 adults with T2D, enrolled in Leumit Health Services, who initiated semaglutide therapy between February 1, 2019, and December 31, 2022. All patients had documented serum 25-hydroxyvitamin D [25(OH)D] levels prior to treatment initiation. Metabolic outcomes- including glycemic control (HbA1c), body mass index (BMI), and lipid profile- were assessed at 12 months. Associations between baseline 25(OH)D levels and metabolic changes were evaluated using multivariable regression models, adjusted for demographic and clinical covariates.

**Results:**

The median baseline 25(OH)D level was 19.3 ng/mL. Compared with patients with 25(OH)D levels <15 ng/mL, those with levels of 15–25 ng/mL and >25 ng/mL exhibited greater reductions in HbA1c [*β* = −0.083, 95% CI (−0.154 to −0.013), *p* = 0.020; and *β* = −0.096, 95% CI (−0.173 to −0.017), *p* = 0.016, respectively] and BMI [*β* = −1.02, 95% CI (−1.46 to −0.58); and *β* = −1.29, 95% CI (−1.77 to −0.80); both *p* < 0.0001]. No association was found with lipid profile.

**Conclusion:**

Higher baseline 25(OH)D levels are independently associated with improved glycemic and weight loss responses to semaglutide in individuals with T2D. Prospective trials are warranted to explore whether vitamin D supplementation may potentiate semaglutide’s metabolic effects.

## Highlights

- Why did we undertake this study?

   Semaglutide is effective in T2D; the influence of baseline 25(OH)D status on treatment response is ill defined.

- What is the specific question we wanted to answer?

   Assessed whether baseline 25(OH)D levels predict glycemic and weight loss responses to semaglutide.

- What did we find?

   Higher 25(OH)D levels were associated with greater reductions in HbA1c and BMI after 12 months of semaglutide.

- What are the implications of our findings?

   25(OH)D may serve as a biomarker for GLP-1RA response and guide metabolic treatment strategies in T2D.

## Introduction

Semaglutide, a glucagon-like peptide-1 (GLP-1) receptor agonist, was initially approved for the treatment of type 2 diabetes mellitus (T2DM) (1) due to its superior glucose-lowering efficacy compared to oral antidiabetic agents, with a favorable safety profile regarding hypoglycemia risk (2–5). In June 2021, the U.S. Food and Drug Administration (FDA) approved higher doses of semaglutide for long-term weight management, marking the first pharmacologic advancement in obesity treatment since 2014.

Semaglutide exerts its effects through multiple mechanisms, including delayed gastric emptying, appetite suppression, enhanced glucose-stimulated insulin secretion from pancreatic *β*-cells, and reduced glucagon secretion. In individuals with overweight and obese, semaglutide induces substantial and sustained weight reduction, with an average decrease of approximately 14.9% of baseline body weight (6).

The pathophysiology of T2DM varies across racial and ethnic groups. For example, T2DM in Caucasian populations is primarily characterized by obesity and insulin resistance, whereas in East Asians, including the Japanese, early-onset *β*-cell dysfunction and insulin deficiency predominate (7, 8). Consequently, the glycemic response to semaglutide is not uniform across populations. Notably, semaglutide predominantly enhances first-phase insulin secretion, an effect that may be particularly beneficial in individuals with insulin deficiency (9, 10). Several blinded, controlled trials have demonstrated superior glycemic control with semaglutide in Asian populations, reinforcing the significance of insulin resistance versus insulin deficiency in determining its net metabolic effects (7, 8, 11–13).

While genetic and ethnic determinants of semaglutide response are largely non-modifiable, certain adjunctive interventions may optimize its therapeutic effects. Among these, vitamin D has emerged as a potential modulator of molecular pathways involved in metabolic regulation. In regard to diabetes control, vitamin D is a key immunomodulatory factor, and preclinical studies have demonstrated its protective effects against *β*-cell dysfunction in diabetes. In murine models, early vitamin D administration mitigated the inflammatory insults responsible for β-cell destruction, thereby preventing autoimmune diabetes onset (14). These findings are supported by human studies in prediabetic and newly diagnosed T2DM individuals, in whom long-term vitamin D supplementation (6 months) significantly improved peripheral insulin sensitivity and β-cell function, potentially delaying diabetes progression (15).

Obesity has long been linked to vitamin D deficiency. While the role of vitamin D in weight loss remains debated, a recent meta-analysis highlighted its potential clinical efficacy in optimizing weight reduction (16). Specifically, 3 months of vitamin D supplementation in obese women significantly augmented weight loss and improved metabolic parameters (17).

Given the high prevalence of vitamin D deficiency, particularly among individuals with obesity and T2DM, supplementation may offer metabolic benefits.

To date, the relationship between vitamin D status and the metabolic effects of semaglutide has not been well characterized. In this retrospective longitudinal study, we aimed to investigate the association between baseline 25(OH)D levels and semaglutide-induced glycemic control and weight loss in an Israeli T2DM population.

## Materials and methods

### Study population

We conducted a population-based study among adult members of Leumit Health Services (LHS), a large Israeli nationwide health maintenance organization (HMO), which provides health services to nearly 730,000 members. LHS has a comprehensive computerized database, continuously updated regarding the demographics, medical diagnoses and clinic visits, hospitalizations, and laboratory tests of insured members.

The socio-economic status (SES) was defined according to the home address. The Israeli Central Bureau of Statistics categorizes all cities and settlements into 20 SES levels. Classification at levels 1–9 is considered low–medium SES, while levels 10–20 represent the medium–high SES. Ethnicity was also defined according to the home address of the HMO members, and categorized into three groups: general population, ultra-orthodox Jews, and Arabs.

All LHS members have identical health insurance coverage and access to healthcare services. Relevant diagnoses are entered or updated according to the International Classification of Diseases 9th revision (ICD-9). The validity of chronic diagnoses in the registry has been previously established. The study population included all LHS members aged 18 or older, with T2DM who were prescribed semaglutide since 2019 and fulfilled the following criteria:

- Consumed semaglutide regularly for at least 6 months.
- Have at least one plasma 25(OH)D level prior to semaglutide initiation.

Only patients aged ≥18 years with a definite diagnosis of T2DM (recorded in the diabetes mellitus registry) were included in the study. T2DM was defined according to the American Diabetes Association classification (26). The exclusion criteria were as follows: (1) missing serum 25[OH]D data; (2) pregnant or lactating females.

Baseline medical conditions including obesity, T2DM, hypertension, asthma, chronic obstructive pulmonary disease, ischemic heart disease, the presence of malignancy, and chronic kidney disease, were recorded. Obesity was defined as BMI > 30 kg/m2. According to LHS guidelines, 25(OH)D tests were collected after overnight fasting and transported on ice to the central laboratory for processing within 4 h of collection using the DiaSorin Chemiluminescence assay (18–21). For categorization of 25(OH)D levels, we calculated the median value and divided the patients into three categories: <15 ng/mL, 15–25 ng/mL, and > 25 ng/mL. The study protocol was approved by the LHS Institutional Review Board (13-21-LEU).

## Statistical analysis

Descriptive statistics, including mean, standard deviation, median, and percentiles, were reported for all study parameters. Group differences were assessed using the *t*-test for continuous variables and Fisher’s exact test for categorical variables. To evaluate the impact of 25(OH)D level categories on changes in HbA1c, BMI, and LDL cholesterol following GLP-1 treatment, linear regression models were fitted with 25(OH)D category as the main explanatory variable. Models were adjusted for age, gender, and the last recorded values of HbA1c, BMI, and LDL cholesterol before treatment initiation.

Variables included in the multivariate analysis were selected based on their statistical significance in univariate analyses. A *p*-value < 0.05 was considered statistically significant. All statistical analyses were conducted using R version 4.4.0.

### Data collection

General demographic information, including age, sex, smoking status, duration of diabetes, and family history of diabetes were collected. Body mass index (BMI) was calculated as weight divided by height squared.

The laboratory measurements collected in this study included: serum 25(OH)D, albumin, triglycerides, total cholesterol, high-density lipoprotein cholesterol (HDL-C), low-density lipoprotein cholesterol (LDL-C), fasting plasma glucose, glycated hemoglobin (HbA1c), and serum creatinine. Notably, serum 25(OH)D concentration was determined by the DiaSorin chemiluminescence assay (18–21) and the detection limit was <10.5 nmol/L. Estimated glomerular filtration rate (eGFR) was calculated using the abbreviated Modification of Diet in Renal Disease (MDRD) equation: 186 × (serum creatinine) − 1.154 × (age) − 0.203 × (0.742 if female).

In addition, diabetic complications (i.e., DR, DKD, DFU, diabetic peripheral neuropathy [DPN]) and related comorbidities (i.e., hypertension, dyslipidemia, coronary heart disease, cerebrovascular disease) were also evaluated.

Subjects were divided into three groups by 25(OH)D level (i.e., <15 ng/mL; 15–25 ng/mL; and >25 ng/mL). Participants were also categorized into four groups based on the levels of BMI: underweight (<18.5 kg/m^2^), normal weight (18.5–24.9 kg/m^2^), overweight (25.0–29.9 kg/m^2^), and obese (≥30.0 kg/m^2^). Glycemic control was classified based on HbA1c levels.

## Results

During the study period, 5,384 individuals with semaglutide-treated T2DM and available baseline plasma 25(OH)D measurements were identified. The demographic and clinical characteristics of the study population are summarized in Table 1. Notably, the median time interval between 25(OH)D assessment and semaglutide initiation was 3.5 months (IQR: 1–7 months).

**Table 1:** Patients’ characteristics.

Patients’ characteristics	[25(OH)D] < 15 ng/mL	[25(OH)D] 15–25 ng/mL	[25(OH)D] > 25 ng/mL	*p* value
Number	1,757	2,109	1,518	
Age (years)	58.0 [51.0;64.0]	61.0 [54.0;68.0]	64.0 [57.0;69.0]	3.8E−51
Female: Male	1,049 (59.7%)	1,080 (51.2%)	849 (55.9%)	
Smoking status	387 (22.05%)	406 (19.29%)	223 (14.69%)	
Ethnic group (Arab)	720 (40.98%)	363 (17.21%)	214 (14.10%)	
Socio-economic status (1-20)	7.00 [5.00;11.00]	10.00 [7.00;12.00]	10.00 [7.00;12.00]	2.8E−48
No hypertension	693 (39.5%)	805 (38.2%)	640 (42.2%)	
<18.5 Underweight	0 (0.000%)	2 (0.095%)	0 (0.000%)	
18.5–24.9 Normal	17 (0.978%)	39 (1.855%)	43 (2.840%)	
25–29.9 Overweight	290 (16.676%)	436 (20.742%)	392 (25.892%)	
> = 30 Obese	1,432 (82.346%)	1,625 (77.307%)	1,079 (71.268%)	
Macroalbuminuria	198 (11.27%)	160 (7.59%)	77 (5.07%)	
Microalbuminuria	703 (40.0%)	707 (33.5%)	451 (29.7%)	
eGFR category				
G1 (Normal)	1,111 (63.23%)	1,151 (54.58%)	753 (49.60%)	
G2 60–89	479 (27.26%)	754 (35.75%)	604 (39.79%)	
G3a 45–59	83 (4.72%)	125 (5.93%)	115 (7.58%)	
G3b 30–44	52 (2.96%)	59 (2.80%)	38 (2.50%)	
G4 15–29	25 (1.42%)	16 (0.76%)	5 (0.33%)	
G5 < 15	7 (0.40%)	4 (0.19%)	3 (0.20%)	
Hemoglobin A1c (%)	7.70 [6.80; 9.00]	7.50 [6.60; 8.50]	7.20 [6.50; 8.07]	3.45E−27
0–6.5	280 (15.95%)	393 (18.63%)	368 (24.24%)	
6.5–8	684 (38.97%)	922 (43.72%)	736 (48.48%)	
8–10	540 (30.77%)	622 (29.49%)	334 (22.00%)	
10+	251 (14.30%)	172 (8.16%)	80 (5.27%)	
Coronary bypass	64 (3.64%)	86 (4.08%)	74 (4.87%)	
Celiac	1 (0.057%)	6 (0.284%)	1 (0.066%)	
Congestive heart failure	100 (5.69%)	108 (5.12%)	68 (4.48%)	
Chronic kidney disease	82 (4.67%)	109 (5.17%)	92 (6.06%)	
COPD	229 (13.03%)	228 (10.81%)	151 (9.95%)	
Cerebral event	97 (5.52%)	94 (4.46%)	88 (5.80%)	
Diabetes end organ damage	672 (38.2%)	751 (35.6%)	566 (37.3%)	
Dyslipidemia	1,227 (69.8%)	1,596 (75.7%)	1,191 (78.5%)	
Hypertension	1,046 (59.5%)	1,392 (66.0%)	1,055 (69.5%)	
Hypothyroidism	145 (8.25%)	184 (8.72%)	171 (11.26%)	
Ischemic heart disease	291 (16.6%)	396 (18.8%)	309 (20.4%)	
Myocardial infarction	133 (7.57%)	162 (7.68%)	100 (6.59%)	
Obstructive sleep apnea	183 (10.4%)	276 (13.1%)	202 (13.3%)	
Album./Creat.Ratio	19.80 [7.79;77.05]	15.77 [6.58;51.64]	13.32 [6.28; 39.64]	1.8E−11
Glucose (mg/dL)	155.2 [126.9;199.4]	145.5 [122.8;181.8]	140.6 [118.6; 167.8]	3.83E−20
HDL Cholesterol (mg/dL)	44.0 [37.0;51.0]	44.0 [37.0;52.0]	44.0 [38.0; 51.0]	0.271648
Triglycerides (mg/dL)	169 [124;246]	166 [118;233]	150 [113; 201]	6.48E−13
LDL Cholesterol (mg/dL)	103.0 [77.2;133.0]	98.0 [74.0;128.0]	88.0 [67.2; 115.0]	1.42E−20

Baseline characteristics of the study population. GFR, glomerular filtration rate; COPD, chronic obstructive pulmonary disease; LDL, low density lipoprotein; HDL, high density lipoprotein.

**Figure 1:**
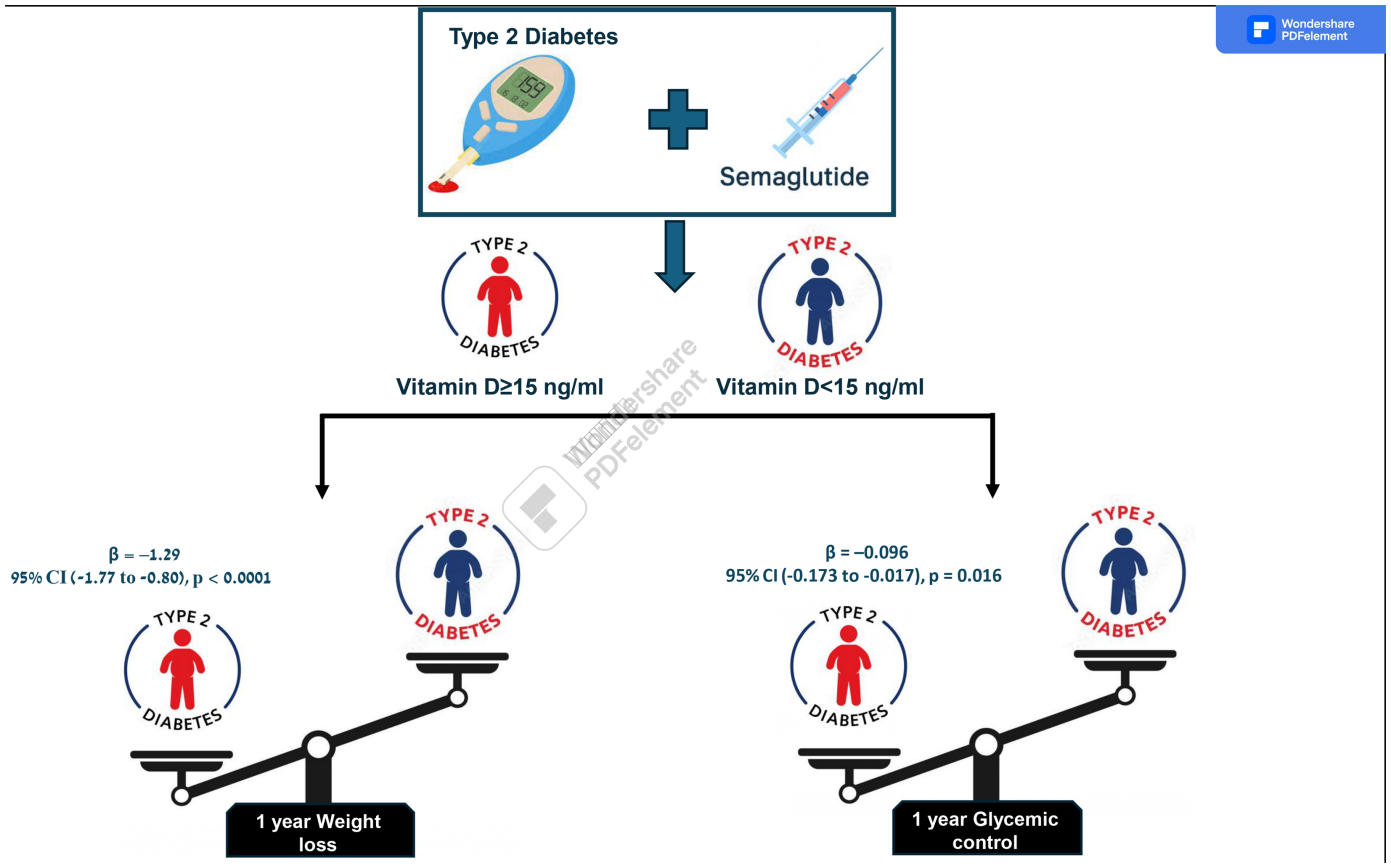
This figure illustrates the distribution of baseline serum 25-hydroxyvitamin D [25(OH)D] levels among all participants prior to GLP-1 treatment initiation. The plot shows a unimodal distribution with a peak between 15 and 25 ng/mL, reflecting the range of vitamin D status in the study population.

Figure 1 displays the distribution of 25(OH)D levels before GLP-1 initiation.

Individuals with higher baseline 25(OH)D levels were significantly older and had a higher prevalence of hypertension, chronic kidney disease (CKD), dyslipidemia, hypothyroidism, prior coronary artery bypass grafting (CABG), ischemic heart disease, and obstructive sleep apnea. Conversely, they exhibited a lower prevalence of microalbuminuria, congestive heart failure, and chronic obstructive pulmonary disease (COPD).

At baseline, a significant inverse association was observed between 25(OH)D levels and key metabolic parameters. Specifically, body mass index (BMI) declined incrementally across increasing 25(OH)D categories [median (IQR)]: <15 ng/mL, 34.0 (30.9–38.6); 15–25 ng/mL, 33.0 (30.1–37.2); >25 ng/mL, 32.1 (29.4–36.2); (*p* < 0.001). A similar trend was noted for glycated hemoglobin (HbA1c) levels [median (IQR)]: <15 ng/mL, 7.70 (6.80–9.00); 15–25 ng/mL, 7.50 (6.60–8.50); >25 ng/mL, 7.20 (6.50–8.07); (*p* < 0.001).

Furthermore, low-density lipoprotein cholesterol (LDL-c) levels also demonstrated a stepwise decrease with higher 25(OH)D levels [median (IQR)]: <15 ng/mL, 103.0 (77.2–133.0); 15–25 ng/mL, 98.0 (74.0–128.0); >25 ng/mL, 88.0 (67.2–115.0); (*p* < 0.001).

These findings suggest that higher baseline 25(OH)D levels are associated with a more favorable metabolic profile prior to semaglutide initiation.

### Weight change

A significant dose–response relationship was observed between baseline 25(OH)D status and weight loss outcomes following 1 year of semaglutide treatment. Participants were stratified into three distinct categories based on 25(OH)D levels. Results showed a progressively greater weight loss with higher baseline 25(OH)D levels compared to the lowest category (<15 ng/mL) [estimate (SE)]: 15–25 ng/mL, −1.02 (*p* = 0.008); and >25 ng/mL, −1.3 (*p* < 0.001).

### Glycemic control

In terms of glycemic control, both higher baseline 25(OH)D categories demonstrated a robust association with improved outcomes [estimate (SE)]: 15–25 ng/mL, −0.083 (0.03) (*p* = 0.02); and >25 ng/mL, −0.095 (0.04) (*p* = 0.017).

### Lipid profile

We found no significant correlation between the prespecified 25(OH)D categories and lipid profile modification.

Table 2 presents the associations between baseline serum 25(OH)D levels and changes in BMI, LDL cholesterol, and HbA1c after 12 months of GLP-1 treatment as assessed by linear regression models.

**Table 2:** Association between baseline 25(OH)D levels and changes in BMI, LDL cholesterol, and HbA1c after 12 months of GLP-1 treatment.

Model target variable	[25(OH)D] category	Regression coefficient	95% confidence interval	*p*-value
BMI difference after 12 months	<15 ng/mL	Ref.		
BMI difference after 12 months	15–25 ng/mL	−1.02	−1.463 to −0.58	<0.0001
BMI difference after 12 months	>25 ng/mL	−1.29	−1.77 to −0.8	<0.0001
LDL Cholesterol difference after 12 months	<15 ng/mL	Ref.		
LDL Cholesterol difference after 12 months	15–25 ng/mL	1.943	−0.105 to 3.991	0.062
LDL Cholesterol difference after 12 months	>25 ng/mL	0.174	−2.082 to 2.430	0.879
Hemoglobin A1c (%) difference after 12 months	<15 ng/mL	Ref.		
Hemoglobin A1c (%) difference after 12 months	15–25 ng/mL	−0.083	−0.154 to −0.013	0.020
Hemoglobin A1c (%) difference after 12 months	>25 ng/mL	−0.096	−0.173 to −0.017	0.016

Linear regression models were used to estimate the association between baseline serum 25-hydroxyvitamin D [25(OH)D] categories and changes in BMI (kg/m^2^), LDL cholesterol (mg/dL), and HbA1c (%) after 12 months of GLP-1 treatment. Models were adjusted for age, gender, diabetes status, and baseline levels of each outcome variable. The reference category was <15 ng/mL for 25(OH)D. Regression coefficients represent the adjusted mean difference in outcome compared to the reference group. CI, confidence interval.

All results are adjusted for covariate differences, including age, gender, diabetes status, and the baseline value of each respective outcome (BMI, LDL cholesterol, or HbA1c) prior to treatment initiation. Compared to individuals with 25(OH)D levels <15 ng/mL, those with levels of 15–25 ng/mL and >25 ng/mL experienced significantly greater reductions in BMI [*β* = −1.02, 95% CI (−1.46 to −0.58), *p* < 0.0001; and *β* = −1.29, 95% CI (−1.77 to −0.80), *p* < 0.0001, respectively]. For HbA1c, both the 15–25 ng/mL and >25 ng/mL groups showed modest but statistically significant reductions compared to the reference group [*β* = −0.083, 95% CI (−0.154 to −0.013), *p* = 0.020; and *β* = −0.096, 95% CI (−0.173 to −0.017), *p* = 0.016, respectively]. No statistically significant differences in LDL cholesterol change were observed across 25(OH)D categories. These findings suggest that higher baseline 25(OH)D levels may be associated with greater improvements in weight and glycemic control following GLP-1 treatment, independent of baseline demographic and clinical differences.

## Discussion

The global prevalence of obesity is surging at an unprecedented rate, emerging as a major public health crisis and a key driver of metabolic disorders, including T2DM. The relationship between obesity and T2DM is well established and multifaceted. Obesity not only significantly increases the risk of developing T2DM but also exacerbates disease severity, with obese individuals being approximately seven times more likely to develop diabetes than those with a normal body weight (22). Excess fat serves as the primary modifiable risk factor for both the onset and progression of T2DM, underscoring its central role in the pathophysiology of the disease. Given the rising global prevalence of obesity and diabetes, there is an urgent need for innovative pharmacological strategies to effectively address the intertwined epidemic of “diabesity.”

In recent years, the glucagon-like peptide-1 (GLP-1) receptor agonist semaglutide has emerged as a highly effective factor for improving glycemic control, facilitating substantial weight loss, and reducing cardiovascular risk. Despite these advancements, diabesity management remains a multifaceted endeavor requiring the integration of multiple tools and factors. One emerging complementary approach to optimize metabolic outcomes is the adjunctive use of vitamin D supplementation, which may serve as a complementary intervention to enhance both glycemic control and weight management (23).

Our study revealed a robust positive association between baseline 25(OH)D levels and semaglutide-induced weight loss in diabetic patients, illuminating a novel area of interest in metabolic research. This relationship suggests that vitamin D, a key regulator of calcium homeostasis and bone metabolism, may have a broader role in energy regulation and weight loss when combined with the GLP-1 receptor agonist, semaglutide. The observed synergistic effect between semaglutide and 25(OH)D may be attributed to vitamin D’s established roles in modulating insulin sensitivity and reducing systemic inflammation. Vitamin D’s effect on insulin sensitivity is mediated via the expression of the vitamin D receptor on pancreatic *β*-cells, which improves insulin secretion and glucose uptake (24). Moreover, vitamin D suppresses pro-inflammatory cytokines including TNF-*α* and IL-6, both implicated in obesity-associated insulin resistance (25) These mechanisms are biologically concordant with the established metabolic effects of semaglutide, which include enhancing insulin secretion, reducing glucagon levels, and promoting satiety through central hypothalamic signaling (26). In addition, vitamin D’s potential impact on lipid metabolism may enhance semaglutide’s effects on weight loss (27). Evidence suggests that vitamin D modulates peroxisome proliferator-activated receptor-*γ* activity, which influences adipocyte differentiation and lipid storage (28). The synergy between these pathways might explain the enhanced weight-loss outcomes observed in patients with higher 25(OH)D levels.

Notwithstanding the promising association between semaglutide and 25(OH)D, the role of vitamin D in weight control remains a subject of considerable debate. A meta-analysis by Bassatne et al. (29) found limited evidence supporting a causal role of vitamin D supplementation in weight loss across diverse populations. These findings contrast with more favorable results from a previous study demonstrating significant weight control achieved after 6 weeks of vitamin D supplementation (30). The above discrepancy highlights the complex interaction between genetic, environmental, and pharmacologic factors influencing the net metabolic response.

Beyond weight control, our findings also indicate significant associations between 25(OH)D levels and improvements in glycemic control. Accumulating evidence suggests that vitamin D’s effect on glucose metabolism likely complements semaglutide’s mechanisms, particularly in enhancing insulin sensitivity and reducing fasting glucose levels. Several studies suggest that adequate levels of 25(OH)D are associated with better glucose control, particularly in individuals with insulin resistance or T2DM (23, 31, 32).

In terms of lipid modification, we found no association between 25(OH)D categorization and lipid reduction. Although some studies pointed out that vitamin D supplementation ameliorated triglycerides and LDL cholesterol levels (33, 34), several high-quality studies (35–37) reported no significant lipid modification of 25(OH)D. We believe that the null effect on lipids is partially attributed to low 25(OH)D levels in the majority of our cohort.

### Study limitations

This study has several limitations. First, its retrospective observational design precludes causal inference, and the findings should be considered associative and hypothesis-generating. Information on vitamin D supplementation was not available and given that such supplements are commonly obtained over the counter, intake could not be reliably assessed. Second, the interval between vitamin D measurement and semaglutide initiation was not included in the analysis, which may introduce exposure misclassification. However, this is an inherent limitation of large real-world datasets, and such misclassification is likely non-differential, biasing results toward the null. Notably, seasonal variability is unlikely to fully account for the findings, as vitamin D deficiency has been shown to be prevalent across ages, sexes, and seasons in Israel (38). Finally, residual confounding cannot be excluded. Variations in dietary habits, physical activity, sun exposure, the number and kind of glucose-lowering and antihyperlipidemic drugs, and the dosage of semaglutide therapy are out of the scope of our study and could have influenced the observed associations.

### Clinical implications

Our findings underscore a plausible importance of vitamin D status in diabetic patients receiving semaglutide therapy. While causation cannot yet be established, vitamin D status may serve as a biomarker of metabolic response to semaglutide. Given the high prevalence of vitamin D deficiency among diabetic and obese populations, routine screening and supplementation may represent a low-cost intervention to maximize the effectiveness of semaglutide therapy and improve therapeutic outcomes.

Future research should aim to explore causative relationships/ mechanisms from observational associations linking 25(OH)D to weight loss and metabolic health. Prospective randomized controlled trials should investigate the impact of vitamin D supplementation in combination with GLP-1 receptor agonists, with a focus on dose–response relationships and long-term metabolic outcomes.

## Conclusion

In conclusion, higher baseline 25(OH)D levels are associated with improved glycemic control and weight loss following semaglutide therapy in patients with T2DM. These findings support a potential role of vitamin D status as a clinically relevant biomarker of treatment response. However, prospective studies are required to determine whether vitamin D supplementation can enhance the metabolic efficacy of GLP-1 receptor agonists.

## Data Availability

The raw data supporting the conclusions of this article will be made available by the authors, without undue reservation.
